# The Extension of Vice

**Published:** 1883-08

**Authors:** 


### THE EXTENSION OF VICE.

Professor J. Edwards Smith has devoted a year to the study and discovery of adulterations in homœopathic medicines! When adulteration strikes the attenuated fabric of the *sim. sim. cur.* materia medica, we may well believe that vice reaches every fibre of our social system.—*Medical Record.*

